# Attosecond Momentum-Resolved Resonant Inelastic X‑ray Scattering for Imaging Coupled Electron–Hole Dynamics

**DOI:** 10.1021/acs.jpclett.6c00998

**Published:** 2026-05-29

**Authors:** Maksim Radionov, Daria Popova-Gorelova

**Affiliations:** † Institute of Physics, 38871 Brandenburg University of Technology Cottbus-Senftenberg, Erich-Weinert-Straße 1, 03046 Cottbus, Germany; ‡ I. Institute for Theoretical Physics and Centre for Free-Electron Laser Science, Universität Hamburg, Notkestr. 9, 22607 Hamburg, Germany

## Abstract

Improving our understanding of electron dynamics is essential for advancing energy transfer, optoelectronics, light-harvesting systems, and quantum computing. Recent developments in attosecond X-ray sources provide the fundamental possibility of observing these dynamics with atomic-scale resolution. However, linking a time-resolved signal to dynamics is challenging due to the broad bandwidth of an attosecond probe pulse. This makes exploring the capabilities of different attosecond imaging techniques crucial. Here, we propose attosecond momentum-resolved resonant inelastic X-ray scattering as a prominent technique for tracking ultrafast dynamics. We demonstrate that the scattering signal contains information about the instantaneous distribution of charge density across the scattering atoms. To illustrate this, we consider scattering from an α-sexithiophene molecule, in which coupled electron–hole dynamics are excited.

## Graphical abstract

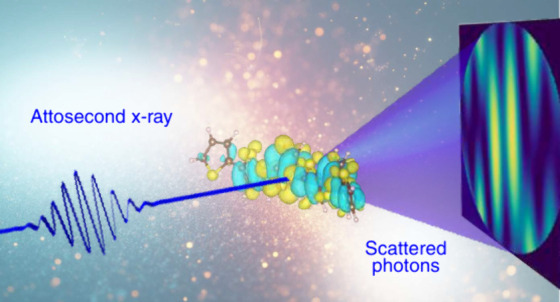

The absorption of an optical or an ultraviolet photon can lead to the neutral excitation of a molecule driving the motion of a charge density.^1^ This motion plays a crucial role in energy transfer,^2^ optoelectronics,^3–5^ photoelectrochemical sensing,^6–10^ quantum computing,^11–15^ and for light-harvesting systems.^16,17^ The time scales of electron dynamics range from sub- to few femtoseconds, and the relevant spatial scales are interatomic distances ranging from Ångstrom to nanometers. The direct observation of electron dynamics requires a combination of attosecond temporal and atomic spatial resolution.^18,19^

Attosecond pump–probe spectroscopy has matured over the past two decades providing the temporal resolution necessary to observe electron dynamics using spectroscopy techniques.^20–25^ Attosecond imaging requires going beyond spectroscopy and the application of scattering and diffraction techniques, which has recently become possible at free-electron laser sources.^26–30^ Attosecond imaging using a pump–probe scheme remains challenging, but efforts are being made to improve the control and stability of attosecond X-ray experiments.^31–33^ Attosecond imaging poses an additional challenge in interpreting signals due to the broad bandwidth of attosecond pulses and the interaction of light with nonstationary electron states. In order to guide experimental developments, it is necessary to investigate and propose beneficial schemes for imaging electronic motion using broad-bandwidth pulses.

X-ray scattering (XRS) with hard X-rays provides (sub)­nanometer spatial resolution. This process is governed by two terms of the light-matter interaction Hamiltonian, namely, **A** · **p** and **A** ^2^, where **A** is the vector potential of an X-ray field and **p** is the momentum operator.^34^ If an X-ray pulse is resonant with the core excitation energy of a system, the former term dominates, and X-ray scattering is referred to as resonant.^35^ If the X-ray pulse is detuned from any transition, the latter term dominates, and scattering is referred to as nonresonant. Nonresonant XRS^36–38^ and sum-frequency diffraction^39^ have been proposed to follow valence-electron motion, attosecond ring currents,^40^ and conical intersection dynamics.^41^ Hybrid X-ray/electron diffraction schemes have also been suggested to trace coupled electron–nuclear dynamics.^42,43^

The conventional resonant X-ray scattering technique measures the momentum of elastically scattered photons,^35^ while resonant inelastic X-ray scattering (RIXS) is a spectroscopic technique.^44^ Momentum-resolved RIXS combines the strengths of both techniques. The advantage of ultrafast resonant X-ray scattering over nonresonant XRS for attosecond imaging is that it enables the selective enhancement of the scattering contribution from the (quasi)­particles involved in the dynamics.^45,46^ In contrast to nonresonant X-ray scattering, it is possible to induce transitions that are forbidden in the ground state resulting in the suppression of a strong stationary background. Another advantage of resonant X-ray scattering over other imaging techniques is its species and orbital selectivity due to transitions involving core orbitals.^35^ The limitation of momentum-resolved RIXS is that its spatial resolution is bound to the wavelength corresponding to the selected X-ray edge, which is low for light atoms. Therefore, the technique is advantageous for molecules containing heavy elements or extended molecular systems like the ones that are considered for optoelectronic applications,^47,48^ or solid-state systems. RIXS is related to resonant Auger scattering (RAS), since both techniques involve transitions to a core-excited state, but one detects emitted electrons in RAS unlike the photon detection in RIXS.^49^ Ultrafast RAS has also been shown to be sensitive to electronic transformations^50^ and its advantage is that it has inherently higher cross section compared to RIXS.^51^ However, RAS is a spectroscopy technique and does not provide a spatial-resolved information.

Several theoretical works have been developed to describe RIXS^52–54^ and momentum-resolved RIXS^45,46,55,56^ from nonstationary electron systems. It has been demonstrated that an attosecond momentum-resolved resonant X-ray scattering signal is noncentrosymmetric due to microscopic electron currents and cannot be straightforwardly related to the time-dependent electron density.^45,46^ It has been suggested that the centrosymmetric component of the signal correlates with the electron density.^57^ However, this idea has not been elaborated upon further. In this study, we demonstrate how to observe the density of a coupled electron–hole using attosecond momentum-resolved RIXS.

It is necessary to perform imaging experiments on aligned molecules so that the details about the charge distribution within the molecule are not averaged out. This condition comes with an advantage that it allows one to avoid the decrease of scattering intensity with an increasing scattering vector **Q** observed in isolated atoms or randomly oriented molecules, where the signal does not contain a contribution due to the interference between the atoms.^58^ It is possible to prepare aligned molecules in a gas phase^59–62^ or adsorb molecules on surfaces in oriented domains.^63^ If a molecule weakly interacts with a surface, such a system may be considered as an approximation to an ensemble of isolated molecules. Electron dynamics of adsorbed molecules strongly coupled to surfaces are interesting on their own in view of optoelectronic applications^64,65^ and fascinating photoinduced effects such as a concerted molecular rotation.^66,67^ The possibility of femtosecond imaging experiments has been demonstrated both for gas-phase molecules^68–70^ and for molecules on substrates.^66,71,72^

We describe an experiment, in which a pump pulse excited an aligned molecule into a coherent superposition of the excited singlet states |Ψ_*n*_⟩ with a hole in HOMO orbitals and an electron in LUMO orbitals with corresponding eigenenergies *ε*_*n*_ creating an excited state Ψ(t)= $\underset{n\geq 1}{\sum }C_{n}e^{-i\varepsilon_{n}t}\left|\Psi_{n}\right\rangle$ . Modern experimental capabilities make it possible to create such a coherent superposition of molecular excited states.^24,73–79^ An X-ray probe pulse acts on the molecule after a nonzero time delay *t*_*p*_ and does not temporally overlap with the pump pulse. We consider the process in which an X-ray probe pulse is tuned to be close, but below X-ray-edge absorption threshold (see Figure 1(a)). Since it is resonant to a transition of a core electron to energy levels that are occupied in the ground state, the scattering signal is primarily caused by excited molecules. This results in a substantial benefit of this method being unaffected by the background due to stationary electrons. We further assume that the X-ray pulse has a Gaussian-shaped temporal profile with duration τ_*p*_ defined as the full width at the half-maximum and has a bandwidth that is considerably larger than the energy splitting between the excited states involved in the dynamics, but smaller than the energy difference between the ground and the excited states. The first condition is necessary to have a sufficient temporal resolution to resolve the excited-state dynamics. The latter condition is beneficial for the exclusion of the signal due to the interference with the ground state. The interference can appear, because the total wave function $\left|\Psi^{\left(\mathrm{tot}\right)}\right\rangle =$ $C_{0}e^{-i\varepsilon_{0}t_{p}}\left|\Psi_{0}\right\rangle +$ $\sqrt{1-|C_{0}{|}^{2}}\left|\Psi \left(t\right)\right\rangle$ includes a contribution from the ground state |Ψ_0_⟩. *C*_0_ is the corresponding complex expansion coefficient. Our assumption describes the experimental conditions, when a time-resolved signal is sensitive only to the excited-state dynamics and would not be distracted by the interference with the ground state. In Section 2 of the Supporting Information, we derive the scattering probability with these assumptions

1 $$P\left(Q,t_{p}\right)=P_{e}P_{0}\theta \left(n_{Q}\right)\underset{F}{\sum }\frac{1}{\omega_{s}}{\left|\underset{n}{\sum }C_{n}e^{-i\varepsilon_{n}t_{p}}e^{-{\left(\varepsilon_{F}+\omega_{s}-\varepsilon_{n}-\omega_{\mathrm{in}}\right)}^{2}\tau_{p}^{2}/8\ln2}\underset{J}{\sum }e^{iQ\cdot R_{J}}\cdot \frac{\left\langle \Psi_{F}\right|\epsilon_{s}^{*}\cdot \nabla \left|\Psi_{J}\right\rangle \left\langle \Psi_{J}\right|\epsilon_{\mathrm{in}}\cdot \nabla \left|\Psi_{n}\right\rangle }{\left(\omega_{s}+\varepsilon_{F}-\varepsilon_{J}+i\frac{\Gamma }{2}\right)}\right|}^{2}$$

where *P*_e_ = 1 – |*C*_0_|^2^ is the probability that the molecule is in an excited state and *P*_0_ is a constant prefactor. **Q** = **k**_s_ – **k**_in_ is the scattered vector; **k**_in_, ω _in_, and **k**_s_, ω_s_ denote the wave vector and photon energy of incoming and scattered radiation, respectively. **ϵ**_in_ is the polarization of the incoming pulse. Since photons are scattered in various possible directions, they have different polarizations, which leads to an additional dependence of the signal on the direction of the scattered vector **n**_*Q*_ through the emission transition matrix elements. This dependence does not carry relevant information. We factored it out into θ­(**n**_*Q*_) by the application of a reflective polarizer for the polarization **ϵ**_s_, please see ref^46^ for details. |Ψ_*J*_⟩ is the intermediate state with a hole at a localized core orbital at position **R**_*J*_. Its energy is denoted as *ε*_*J*_ and its lifetime broadening as Γ. The first sum runs over all possible final states |Ψ_*F*_⟩ with energies *ε*_*F*_. In the following analysis, we focus on such transitions that result in a scattered photon being close to the incoming photon energy (see Figure 1(a)). In this case, a final state would either coincide with the ground state, one of states involved in the dynamics or some other valence-excited state. Any possible transition is inelastic, since the initial state is a nonstationary state. We use atomic units for this and the following equations.

**Figure 1:**
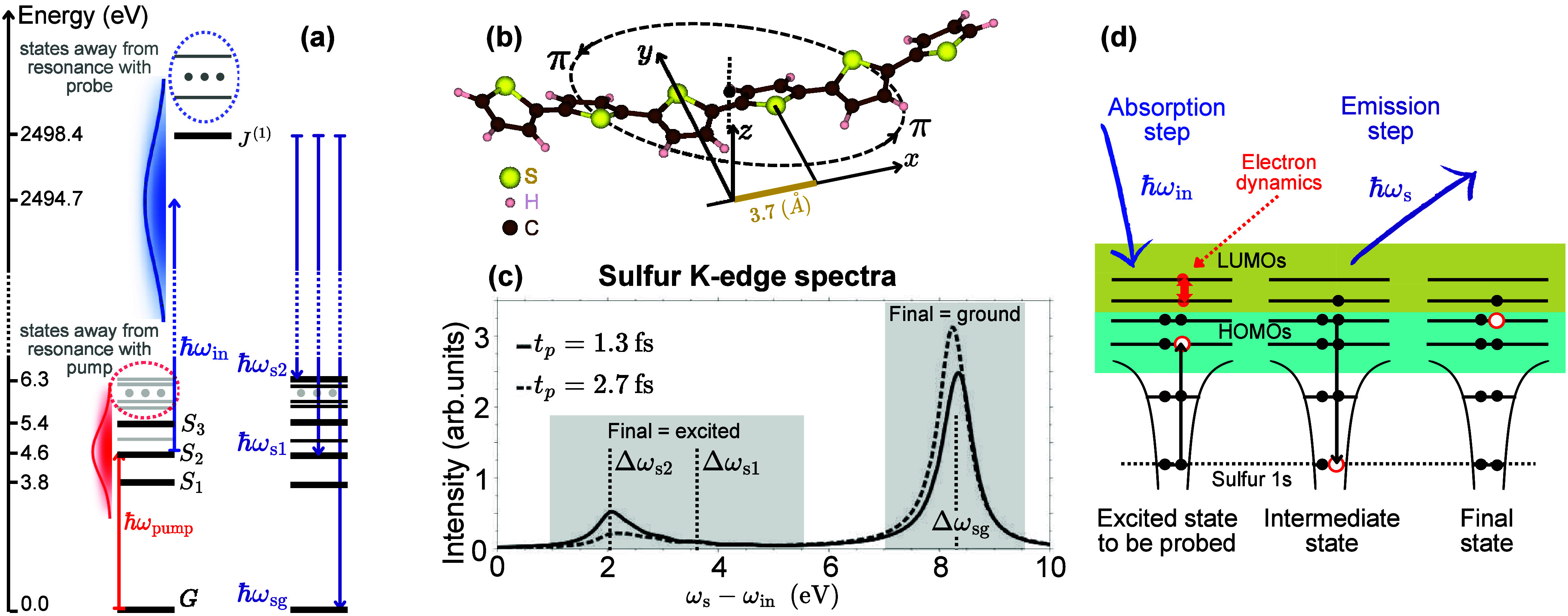
(a) Illustration of attosecond pump–probe experiment. (b) Sexithiophene molecule. (c) Sulfur K-edge spectra of an excited sexithiophene molecule at different time delays (**k**_in_∥*y*, **ϵ**_in_∥*x*). (d) Illustration of states involved in the dynamics and transitions.

We demonstrate the power of attosecond resonant inelastic X-ray scattering to reveal information about coupled electron–hole dynamics by considering scattering on the sexithiophene molecule shown in Figure 1(b). Sexithiophene is noteworthy for optoelectronic applications due to its special optical properties governed by excitonic excited states.^81–83^ The alignment of sexithiophene molecules was achieved on various surfaces.^84–86^ We take into account many-body effects due to electron–hole coupling using the restricted active space configuration interaction (RASCI) method.^87^

We assume that a pump pulse created a coherent superposition of the first three bright excited singlet states with the energies 3.8 eV, 4.6 eV and 5.4 eV at time *t*_*p*_ = 0. We set the ground-state energy to zero. Excited states with higher energies can be excluded from the superposition in the pump process, since they are energetically separated (see Figure 1(a)). We select the coefficients *C*_1_, *C*_2_ and *C*_3_ to be equal to $1/\sqrt{3}$ , which can be achieved by selecting the appropriate pump-pulse parameters. The conclusions of our study are independent of the specific choice of coefficients. We show the difference between ρ­(**r**,*t*_*p*_) and the ground-state electron density ρ_*G*_(**r**) at two different time delays in Figures 2 (a) and (b). We also disentangle the hole and electron contributions to the differential electron density as described in Section 2 of Supporting Information and show them below it.

**Figure 2:**
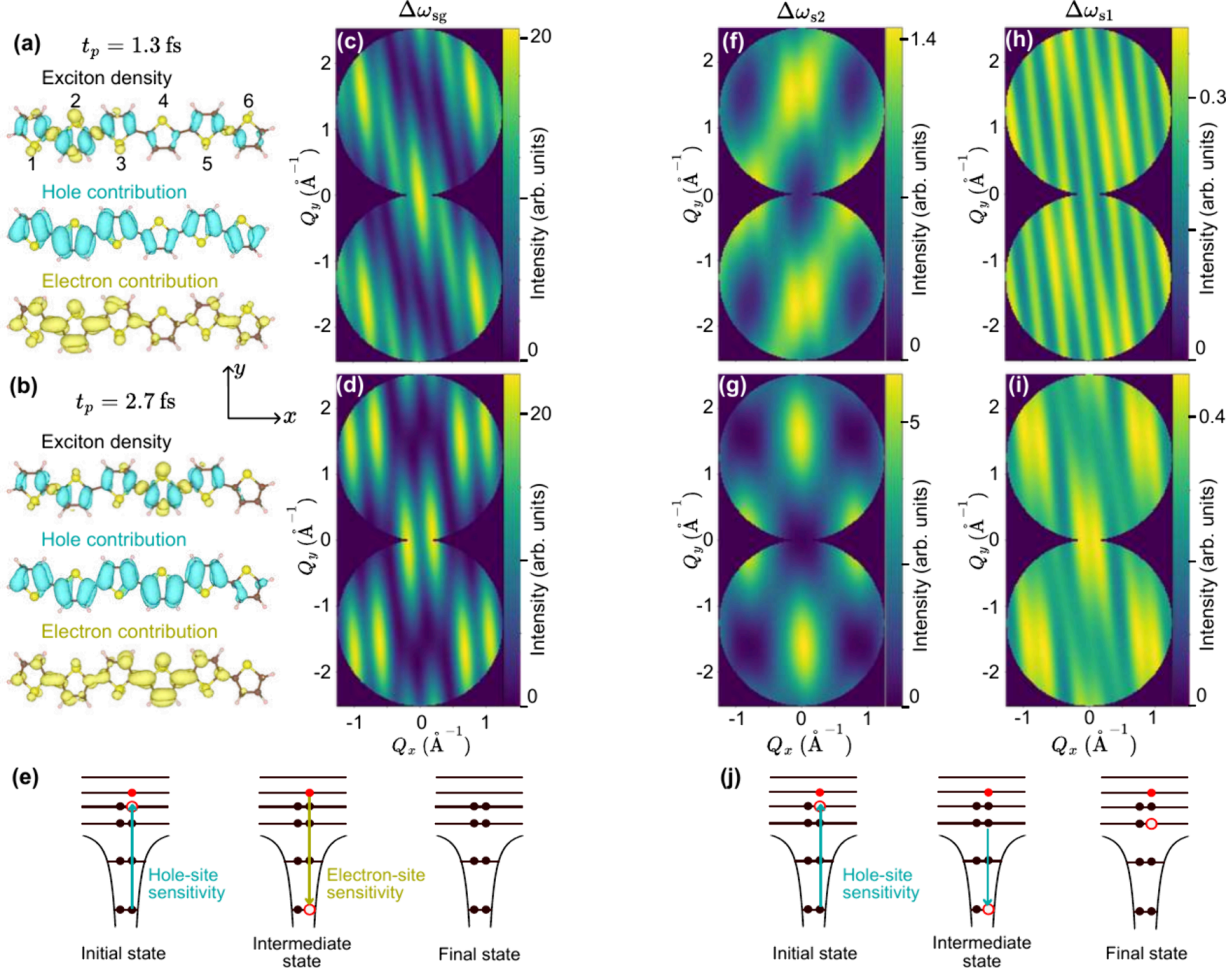
(a) – (b) ρ­(**r**,*t*_*p*_) – ρ_*G*_(**r**), its hole and electron contributions at (a) *t*_*p*_ = 1.3 fs and (b) *t*_*p*_ = 2.7 fs visualized with the VESTA package.^80^ Yellow and cyan isosurfaces correspond to the negatively- and positively charged regions. (c)–(d) The even part of the momentum maps at *Q*_*z*_ = 0, [*P*(*Q_x_*,*Q_y_*,0) + *P*(−*Q_x_*,–*Q_y_*,0)]/(2θ­(**n** *_Q_*)), with the final state being the ground state at (c) *t*_*p*_ = 1.3 fs and (d) *t*_*p*_ = 2.7 fs and (e) illustration of the involved transitions. (f)–(i) The same as (c)–(d), but with the final state being a valence-excited state at (f) ω_s_ – ω_in_ = Δω_s2_ and *t*_*p*_ = 1.3 fs; (g) ω_s_ – ω_in_ = Δω_s2_ and *t*_*p*_ = 2.7 fs; (h) ω_s_ – ω_in_ = Δω_s1_ and *t*_*p*_ = 1.3 fs; and (i) ω_s_ – ω_in_ = Δω_s1_ and *t*_*p*_ = 2.7 fs. (j) Illustration of the scattering process with a final state being a valence-excited state.

We assume that the X-ray probe pulse has a mean photon energy ω_in_ = 2490 eV and a duration τ_*p*_ = 300 as, which corresponds to a bandwidth of 2.6 eV, ***k***_in_∥*y* and **ϵ**_in_∥*x*. This pulse duration is shorter than the shortest beating period of the electron density, which is necessary to achieve precise temporal resolution. However, as shown in the Supporting Information, the signal remains time-dependent for probe–pulse durations of up to 600 as. The assumed X-ray pulse is resonant with transitions from the states |Ψ_1,2,3_⟩ to the intermediate states with a hole in the sulfur 1*s* orbitals. The energies of the core-excited states of sexithiophene vary slightly depending on the location of the core hole, by no more than 120 meV. This forms groups of core-excited states, depending on the character of the excitations in the LUMO orbitals. We set ω_in_ such that transitions to the lowest-energy group of core-excited states dominate, thus facilitating the analysis. The lifetime broadening of sulfur 1*s*-excited states Γ is 0.59 eV.^88^

Figure 1(c) shows momentum-unresolved spectra at two different time delays. The right intensive peak corresponds to emission into a final state that is the ground state. The peak is actually composed of several peaks. The positions of the individual peaks do not vary over time, but the intensity does, which leads to the illusion that the peak shifts. The left broad peak is due to emission with final states being valence excited states. The bandwidth of the individual peaks is determined by both the spectral bandwidth of the probe pulse and the Lorenzian lifetime broadening.

Figures 2 (c), (d), (f)-(i) show the centrosymmetric part of the time- and momentum-resolved RIXS signal, [P(Q)+P(−Q)]/2 , at the three different scattered energies outlined in Figure 1 (c), and at different time delays. This part of the signal follows the electron density, since it involves the same interference terms $\underset{n_{1},n_{2}>n_{1}}{\sum }R\left(C_{n_{2}}^{*}C_{n_{1}}e^{-i\left(\varepsilon_{n_{1}}-\varepsilon_{n_{2}}\right)t_{p}}\right)$ as the density does.^57^ Here, we apply the polarizer for **ϵ**_s_∥*z* and divide the signal by θ­(**n**_*Q*_). The total time- and momentum-resolved RIXS signal shown in the supplementary Figure S2 is not centrosymmetric due to the presence of currents.^45^ Figures S3 and S4 show the signal obtained without applying the polarizer and dividing by θ­(**n**_*Q*_) for the same **Q** plane as in Figure 2 and for a fixed **k**_in_, correspondingly. They all have prominent time-dependent features described below, but these features become less pronounced. Therefore, while the application of the polarizer, division by θ­(**n**_*Q*_) and fixing *Q*_*z*_ = 0 are desirable for direct linking to a density, they are not necessary.

Since the intermediate states have localized holes on sulfur atoms, only sulfur atoms contribute as scattering atoms, which means that the scattering signal is sensitive to the electron distribution in the vicinity of sulfur atoms. The **Q**-dependent part of the signal is a combination of periodic functions with periods 1/λ_*ik*_, where λ_*ik*_ = |**R**_*i*_ – **R**_*k*_|/2π with *i* and *k* denoting different sulfur atoms. The spatial resolution is sufficient to resolve oscillations due to nearest-neighbor sulfur atoms separated by 4.3 Å.

The scattering signal is sensitive to the excited-state dynamics through the time-dependent absorption amplitudes. During the absorption step of the scattering process, a core electron localized on a sulfur atom *i* fills the delocalized hole. This transition is only possible if the hole density around the atom *i* at the time of the measurement is considerable. Interference fringes in the scattering signal appear, if a pair of atoms scatters. Thus, maxima separated by 1/λ_*ik*_ in the signal indicate that the hole density is simultaneously nonzero around a pair of atoms *i* and *k*. For example, the hole density on the most widely separated atoms 1 and 6 is considerable at time *t*_*p*_ = 1.3 fs (see Figure 2(a)). In contrast, the hole density on the atom 6 is negligible at time *t*_*p*_ = 2.7 fs (see Figure 2(b)). Consequently, the shortest-period oscillations can clearly be observed in all momentum maps at *t*_*p*_ = 1.3 fs, but are not visible in the maps at *t*_*p*_ = 2.7 fs. The scattering signal, thus, encodes information about the hole density at the time of measurement.

It is also relevant to investigate the electron contribution to the excited-state dynamics. This contribution is due to the excited states having electrons distributed in the LUMO states. It turns out that the signal is also sensitive to this electron contribution, if the molecule’s final state is the ground state. This connection is nontrivial, so let us explain it using the independent-particle picture. After the action of the probe pulse, a core electron is excited to a HOMO orbital, while the same LUMO orbital remains occupied after absorption (see Figure 2(e)). During emission, the LUMO electron fills the core hole, placing the system in the ground state. For emission to be possible, the overlap of the LUMO orbital with the unoccupied core-orbital must be considerable. Therefore, both an excited-state hole and electron distribution must be considerable around a scattering atom for scattering to be possible. A λ_*ik*_ oscillation in the corresponding signal reflects the simultaneous presence of an electron–hole pair on atoms *i* and *k*. The hole and electron distributions of optically excited sexithiophene move almost synchronously, enhancing the contrast of the oscillations.

Now, let us consider transitions in which the final state is not the ground state, but rather a valence-excited state. Due to the same absorption step, the signal would still be sensitive to the excited-state hole density. However, the connection to the electron contribution would not hold. We use the independent-particle picture again to illustrate this mechanism (see Figure 2 (j)). For absorption and subsequent emission to a final state being an excited state, all states involved must have the same LUMO orbital being occupied by an electron. This condition excludes some possible final states and the only role of the LUMO electron is now to select possible final states. Emission results from a transition between a HOMO orbital and unoccupied core orbitals. The structure of this HOMO orbital determines whether the **Q** dependence due to the hole distribution around scattering atoms is suppressed or enhanced as can be observed in Figures 2 (f)-(i). Unlike the energetically separated ground state, the valence-excited states are close in energy, making it impossible to disentangle the role of an individual final state in the signal. Nevertheless, analyzing the signal at such scattering energies is advantageous. For scattering with the final state being the ground state, interference fringes are possible, only if both excited-state electron and hole distributions are considerable around an atomic pair. If the signal is now analyzed at a different scattered energy, new interference fringes can occur. This would indicate that a considerable hole distribution exists around some atoms where the electron distribution is negligible.

We introduced a method to extract information about the time-dependent charge density with the momentum-resolved RIXS. The prominent advantage of this technique is the resonant enhancement of the signal from moving particles. Through an in-depth analysis of the signal from a nonstationary electronic system and the transitions involved, and by illustrating the study with accurate many-body calculations, we were able to identify favorable experimental conditions for linking signal features to charge density properties. The signal is a mixture of contributions due to the charge density and to microscopic currents. We demonstrated how the charge density contribution can be separated using a straightforward procedure. Since the process involves localized intermediate states, the signal is sensitive to charge distributions on scattering sites. By analyzing the momentum distribution at different scattered energies, it is possible to identify the sites with the significant distribution of holes, and those that have significant distributions of both holes and electrons. This technique is particularly useful for studying the dynamics of excitons and charge separation, since it provides means to distinguish coupled and decoupled electron–hole dynamics.

## Computational Methods

We take into account many-body effects due to electron–hole coupling using the restricted active space configuration interaction (RASCI) method^87^ implemented in the MOLCAS package.^89^ We calculate eigenstates and eigenenergies of sexithiophene, and use them to calculate the electron density and the scattering signal with eq 1 (see Section 1 of the Supporting Information for further computational details).

## Supplementary Material



## Notes

Computational details (PDF)
Derivation of equations (PDF)
Total scattering signal (PDF)
Signal at different probe–pulse durations (PDF)
