# Supplementary material for: Attosecond Momentum-Resolved Resonant Inelastic X‑ray Scattering for Imaging Coupled Electron–Hole Dynamics
Source: J Phys Chem Lett. 2026 May 29;17(23):6520–7. doi: 10.1021/acs.jpclett.6c00998 (PMC13267081; doi:10.1021/acs.jpclett.6c00998)
Supplement: Supplementary file 1 [file jz6c00998_si_001.pdf]

Supporting Information for "Attosecond momentum-resolved resonant inelastic x-ray scattering for imaging coupled electron-hole dynamics"

Maksim Radionov[1,2] and Daria Popova-Gorelova[1,2]
[1] *Institute of Physics, Brandenburg University of Technology Cottbus-Senftenberg, Erich-Weinert-Straße 1, 03046 Cottbus, Germany and*
[2] *I. Institute for Theoretical Physics and Centre for Free-Electron Laser Science, Universität Hamburg, Notkestr. 9, 22607 Hamburg, Germany*

1. COMPUTATIONAL DETAILS

The calculations of the electronic states of the isolated sexithiophene molecule are performed using the MOLCAS software [1]. The molecular geometry has been taken from a molecular structure database PubChem [2]. To calculate the ground state, we use the Hartree-Fock approach [3] within the ANO-S-VTZP atomic orbital basis set [4]. We use these molecular orbitals without further optimization to obtain the excited states within the restricted active space configuration interaction (RASCI) method [5]. The RASCI method represents the excited state as a sum of configuration state functions (CSFs) [6]. We restrict the expansion to the singly excited CSFs, such that the molecular eigenstate is $|\Psi_n\rangle = \sum\limits_{i,a} c_{ia}^{(n)}|\Phi_i^a\rangle$, where $|\Phi_i^a\rangle$ denotes a CSF with a hole in the ith molecular orbital and an additional electron on the ath molecular orbital. The calculations of the valence-excited states converge with respect to the active space on 100 inactive orbitals, 27 RAS1 orbitals, and 27 RAS3 orbitals [7]. Calculating charge dynamics, we neglect the nuclear motion assuming that the coherence loss is negligible for the considered time scales of few femtoseconds [8–11].

We have compared our results with the available experimental data. In our calculations, the energy difference between the first excited state and the ground state is 3.8 eV. There are no data on the isolated molecules, but there are many experiments on sexithiophene in the solid phase and in various solutions. The first absorption peak appears at approximately 2.3 – 3.0 eV [12–17].

We calculate the core-excited intermediate states using the RASCI with the highly excited states (HEXS) method, following the procedure described in [18]. The first six molecular orbitals are predominantly represented as linear combinations of six sulfur $1s$ basis atomic orbitals. In six different calculations, we put one of these molecular orbitals into the RAS1 subspace. The RAS3 subspace is kept the same as for the valence-excited states (see Fig. S1). Since the core molecular orbitals are delocalized, the core hole in the calculated excited state, J', is also delocalized. The first six core-excited states are nearly degenerate in energy (the energy difference is less than 120 meV). This means that the states J' can be transformed into six states J with a localized core hole. The state J' can then be expressed as a linear combination of the CSFs with a hole in the sulfur $1s$ molecular orbital j' and an electron in an unoccupied orbital b: $|\Psi'_{J'}\rangle = \sum\limits_b c_b^{(J')}|\Phi_{j'}^b\rangle$.

We express the field operators $\hat{\psi}$ and $\hat{\psi}^\dagger$ in the basis of the electron creation $\hat{c}^\dagger$ and annihilation $\hat{c}$ operators and rewrite the transition matrix elements between the valence and sulfur $1s$-excited states in the basis of the CSFs:

$$\langle \Psi'_{J'}|\hat{\psi}^\dagger e^{i\boldsymbol{k}\cdot\boldsymbol{r}}(\boldsymbol{\epsilon}\cdot\boldsymbol{\nabla})\hat{\psi}|\Psi_n\rangle = \sum_{p,q}\sum_{a,i,b} c_b^{(J')^*} c_{ia}^{(n)} \times \langle\Phi_{j'}^b|\hat{c}_p^\dagger \hat{c}_q|\Phi_i^a\rangle\langle\phi_p|e^{i\boldsymbol{k}\cdot\boldsymbol{r}}\boldsymbol{\epsilon}\cdot\boldsymbol{\nabla}|\phi_q\rangle, \tag{S1}$$

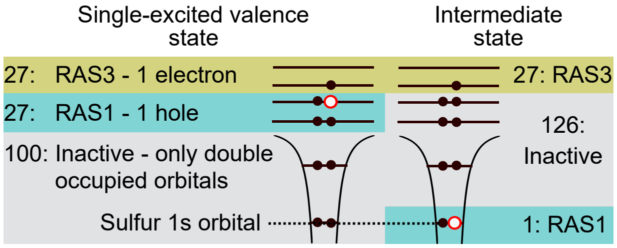

FIG. S1. Restricted active spaces for calculations of the valence-excited and $1s$-excited states.

where the matrix element $\langle\Phi^b_{j'}|\hat{c}^\dagger_p\hat{c}_q|\Phi^a_i\rangle$ is nonzero only if the two configurations differ by at most one occupied orbital. Since the molecular orbital $\phi_{j'}$ corresponds to the sulfur $1s$ orbital, which is doubly occupied in the configuration $|\Phi^a_i\rangle$ and singly occupied in $|\Phi^b_{j'}\rangle$, the annihilation operator $\hat{c}_q$ yields a nonzero value only when $q = j'$. We expand the molecular orbital as a linear combination of the basis atomic orbitals, $|\phi_{j'}\rangle = \sum_{N,o} \Xi_{j',N,o}|\xi_{N,o}\rangle$. Here, o denotes the index of the basis atomic orbital of the atom N. For the first six molecular orbitals j', the coefficients $\Xi_{j',N,o}$ are significant only for strongly localized sulfur $1s$ atomic orbitals. Assuming that the x-ray wavelength is much larger than the spatial extent of the $1s$-orbital leads to the approximation $e^{i\mathbf{k}\cdot\mathbf{r}}\Xi_{j',N,o}|\xi_{N,o}\rangle \approx e^{i\mathbf{k}\cdot\mathbf{R}_N}\Xi_{j',N,o}|\xi_{N,o}\rangle$. The transition matrix element can then be expressed as

$$\begin{aligned}\langle\phi_p|e^{i\boldsymbol{k}\cdot\boldsymbol{r}}(\boldsymbol{\epsilon}\cdot\boldsymbol{\nabla})|\phi_{j'}\rangle &= \sum_{N,o}\Xi_{j',N,o}\langle\phi_p|e^{i\boldsymbol{k}\cdot\boldsymbol{r}}(\boldsymbol{\epsilon}\cdot\boldsymbol{\nabla})|\xi_{N,o}\rangle \\ &\approx \sum_{N,o}\Xi_{j',N,o}e^{i\boldsymbol{k}\cdot\boldsymbol{R}_N}\langle\phi_p|\boldsymbol{\epsilon}\cdot\boldsymbol{\nabla}|\xi_{N,o}\rangle. \end{aligned} \tag{S2}$$

2. EQUATION DERIVATION

The general expression for attosecond momentum-resolved RIXS has been derived in Ref. [19] using the time-dependent second-order perturbation theory and the second quantization formalism. In the present work, we follow the same derivation steps, but (i) do not integrate the signal over the energy window and (ii) do not apply the mean energy approximation for the non-stationary electronic system.

We first show that the scattering probability does not have a background due to the ground state. If the initial state is $|\Psi^{(\mathrm{tot})}\rangle$, the scattering probability can be expressed as a function of the probe pulse parameters ω_{in} and τ_p [19]:

$$\begin{aligned}P(\omega_{\mathrm{in}},\tau_p) = a\sum_{F,s_{\mathrm{s}}}\Bigg| & C_0 e^{-\frac{(\varepsilon_F+\omega_{\mathrm{s}}-\varepsilon_0-\omega_{\mathrm{in}})^2\tau_p^2}{8\ln 2}} b_{0,F} \\ &+\sum_{n\geq 1}\sqrt{1-|C_0|^2}C_n e^{-\frac{(\varepsilon_F+\omega_{\mathrm{s}}-\varepsilon_n-\omega_{\mathrm{in}})^2\tau_p^2}{8\ln 2}} b_{n,F}\Bigg|^2, \end{aligned} \tag{S3}$$

where coefficients a and $b_{n,F}$ are constant coefficients. The first term in the modulus is the contribution of the ground state to the scattering signal. Since, in our case, the energy difference between the ground state and any excited state is much larger than the energy difference between any two excited states, the factor $(\varepsilon_F + \omega_{\mathrm{s}} - \varepsilon_0 - \omega_{\mathrm{in}})$ in the exponent in the first term is considerably larger than that in the other terms, $(\varepsilon_F + \omega_{\mathrm{s}} - \varepsilon_n - \omega_{\mathrm{in}})$, for any $n \geq 1$. Consequently, the exponent in the first term is much smaller than those in the others. For our parameters $\tau_p = 300$ as, $\omega_{\mathrm{in}} = 2490$ eV, it is approximately ten times smaller. Therefore, we neglect the first term and express the scattering probability as:

$$P(\omega_{\mathrm{in}},\tau_p) \approx aP_{\mathrm{e}}\sum_{F,s_{\mathrm{s}}}\left|\sum_{n\geq 1}C_n e^{-\frac{(\varepsilon_F+\omega_{\mathrm{s}}-\varepsilon_n-\omega_{\mathrm{in}})^2\tau_p^2}{8\ln 2}} b_{n,F}\right|^2, \tag{S4}$$

where $P_{\mathrm{e}} = \left(1-|C_0|^2\right)$ is the probability that the system is in an excited state. The contribution of the ground state is now in the pre-factor P_{e}.

Using the approximation in Eq. (S2) for the transition matrix element in Eq. (S1) and the approximation in Eq. (S4), we obtain the following expression for the attosecond momentum-resolved RIXS signal:

$$P(\mathbf{k}_\mathrm{s}, t_p) = P_\mathrm{e}\,\theta(\mathbf{n}_Q) P_0 \sum_F \frac{1}{\omega_\mathrm{s}} \left| \sum_n C_n e^{-i\varepsilon_n t_p} e^{-\frac{(\varepsilon_F + \omega_\mathrm{s} - \varepsilon_n - \omega_\mathrm{in})^2 \tau_p^2}{8\ln 2}} \sum_{J'} \frac{1}{\left(\omega_\mathrm{s} + \varepsilon_F - \varepsilon_{J'} + i\frac{\Gamma}{2}\right)} \right.$$
$$\times \left(\boldsymbol{\epsilon}_\mathrm{s}^* \cdot \sum_{p_1,q_1} \sum_{N_1,o_1} e^{-i\boldsymbol{k}_\mathrm{s}\boldsymbol{R}_{N_1}} \Xi^*_{p_1,N_1,o_1} \langle \xi_{N_1,o_1} | \boldsymbol{\nabla} | \phi_{q_1} \rangle \langle \Psi_F | \hat{c}^\dagger_{p_1} \hat{c}_{q_1} | \Psi'_{J'} \rangle \right)$$
$$\left. \times \left(\boldsymbol{\epsilon}_\mathrm{in} \cdot \sum_{p_2,q_2} \sum_{N_2,o_2} e^{i\boldsymbol{k}_\mathrm{in}\boldsymbol{R}_{N_2}} \Xi_{q_2,N_2,o_2} \langle \phi_{p_2} | \boldsymbol{\nabla} | \xi_{N_2,o_2} \rangle \langle \Psi'_{J'} | \hat{c}^\dagger_{p_2} \hat{c}_{q_2} | \Psi_n \rangle \right) \right|^2, \quad \text{(S5)}$$

where $P_0 = 2\pi^3 I_0 \tau_p^2 / (\ln 2 c V \omega_\mathrm{in}^2)$ with I_0 being the peak intensity of the probe pulse, c – the speed of light, and V – the quantization volume.

As described in Section 1, we represent the state $|\Psi_J\rangle$ with a hole localized on a single sulfur atom j as a linear combination of the calculated intermediate states $|\Psi_J\rangle \approx \sum_{j'} \alpha_{jj'} |\Psi'_{J'}\rangle$. Under this approximation, Eq. (1) in the main text can be obtained from Eq. (S5). We have checked that the scattering probabilities calculated with and without this assumption differ by less than 1%.

During the excitation process, the absorption of an UV photon creates an electron-hole pair. Consequently, the charge density of the excited state $\rho = |\Psi(t)|^2$ differs from that of the ground state ρ_G by one hole in the RAS1 space (HOMOs) and one electron in the RAS3 space (LUMOs). We denote this difference by the exciton density. The exciton density has the contribution due to the LUMOs electron density $\rho_\mathrm{e}(t_p)$ and the HOMOs hole density $\rho_\mathrm{h}(t_p)$, which we disentangle with the procedure below:

$$\rho(t_p, \boldsymbol{r}) - \rho_G(\boldsymbol{r}) = \rho_\mathrm{e}(t_p) + \rho_\mathrm{h}(t_p) = \sum_{m,n} C_m^* C_n e^{i(\varepsilon_m - \varepsilon_n)t_p} \sum_{p,q} \langle \Psi_m | \hat{c}^\dagger_p \hat{c}_q | \Psi_n \rangle \phi_p^*(\boldsymbol{r}) \phi_q(\boldsymbol{r}) - \sum_{p \in \mathrm{HOMOs}} |\phi_p(\boldsymbol{r})|^2,$$

$$\rho_\mathrm{e}(t_p) = 2\,\mathrm{Re}\left(\sum_{m,n \geq m} C_m^* C_n e^{i(\varepsilon_m - \varepsilon_n)t_p} \sum_{p,q \in \mathrm{LUMOs}} \langle \Psi_m | \hat{c}^\dagger_p \hat{c}_q | \Psi_n \rangle \phi_p^*(\boldsymbol{r}) \phi_q(\boldsymbol{r}) \right), \quad \text{(S6)}$$

$$\rho_\mathrm{h}(t_p) = 2\,\mathrm{Re}\left(\sum_{m,n \geq m} C_m^* C_n e^{i(\varepsilon_m - \varepsilon_n)t_p} \sum_{p,q \in \mathrm{HOMOs}} \langle \Psi_m | \hat{c}^\dagger_p \hat{c}_q | \Psi_n \rangle \phi_p^*(\boldsymbol{r}) \phi_q(\boldsymbol{r}) \right) - \sum_{p \in \mathrm{HOMOs}} |\phi_p(\boldsymbol{r})|^2. \quad \text{(S7)}$$

3. TOTAL SIGNAL

Figure S2) shows the total signal $P(Q_x, Q_y, 0)$ divided by $\theta(\mathbf{n}_Q)$. Note that it is not centrosymmetric due to currents. We show its centrosymmetric part in the main text.

Figure S3 shows the centrosymmetric part of the scattering probability $P(Q_x, Q_y, 0)$ divided by $\theta(\mathbf{n}_Q)$ next to the scattering probability not divided by $\theta(\mathbf{n}_Q)$ at two different time delays. It is shown for the case, when the final state coincides with the ground state. The patterns in Figs. S3(a) and S3(c) coincide with the patterns shown in the main text in Figs. 2(c) and 2(d). The red dashed lines indicate that one slice through the data is enough to observe the periodicity in the signal connected to the charge distribution over the molecule.

In the main text, we show the patterns, corresponding to a momentum plane with $Q_z = 0$, which can be achieved only if $\mathbf{k}_\mathrm{in}$ is rotated. However, this is not a necessary condition. Figure S4 shows scattering patterns for a fixed $\mathbf{k}_\mathrm{in} \parallel z$ without the application of the reflection polarizer and division by $\theta(\mathbf{n}_Q)$. The probability is now shown as a function of $\mathbf{k}_\mathrm{s}$ and, therefore, the data is limited by a single circle. The figure demonstrates that the signal preserves periodicities related to the charge distribution within the molecule in this less challenging experimental geometry.

4. PULSE DURATION

The contribution of each eigenstate $|\Psi_n\rangle$ involved in the superposition of the excited states is weighted by a function $G(\varepsilon, \tau_p) = e^{-\frac{(\varepsilon_F + \omega_\mathrm{s} - \varepsilon_n - \omega_\mathrm{in})^2 \tau_p^2}{8\ln 2}}$. This factor decreases with increasing detuning of the transition energy from the photon

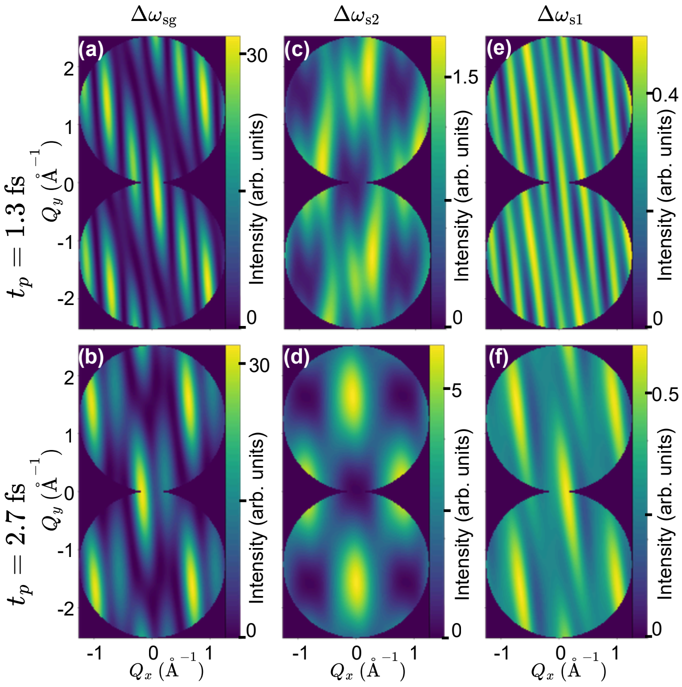

FIG. S2. The momentum maps $P(Q_x, Q_y, 0)/(\theta(\mathbf{n}_Q))$ at $Q_z = 0$ with the final state being the ground state ($\omega_{\rm s} - \omega_{\rm in} = \Delta\omega_{\rm sg}$) at (a) $t_p = 1.3$ fs and (b) $t_p = 2.7$ fs. (c)–(f) The same as (a)–(b), but with the final state being a valence-excited state at (c) $\omega_{\rm s} - \omega_{\rm in} = \Delta\omega_{\rm s2}$ and $t_p = 1.3$ fs; (d) $\omega_{\rm s} - \omega_{\rm in} = \Delta\omega_{\rm s2}$ and $t_p = 2.7$ fs; (e) $\omega_{\rm s} - \omega_{\rm in} = \Delta\omega_{\rm s1}$ and $t_p = 1.3$ fs and (f) $\omega_{\rm s} - \omega_{\rm in} = \Delta\omega_{\rm s1}$ and $t_p = 2.7$ fs. The time delays and scattering energies are chosen to be the same as in the main text. Therefore, panels (a) and (b) can be compared with Figs. 2(c) and 2(d); (c) and (d) – with Figs. 2(f) and 2(g); (e) and (f) – with Figs. 2(h) and 2(i).

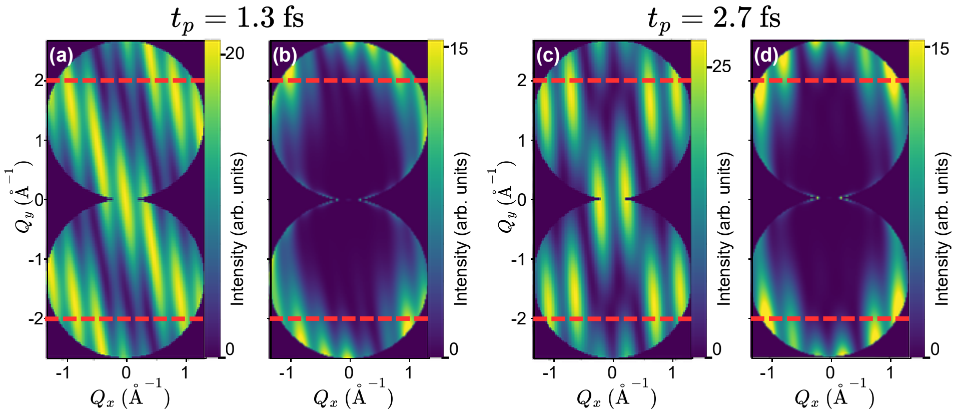

FIG. S3. The symmetric parts of the momentum maps $P\left((Q_x, Q_y, 0), t_p\right)/\theta(\mathbf{n}_Q)$ and $P\left((Q_x, Q_y, 0), t_p\right)$ at $Q_z = 0$ with the final state being the ground state ($\omega_{\rm s} - \omega_{\rm in} = \Delta\omega_{\rm sg}$) are compared at pump-probe delays (a)-(b) $t_p = 1.3$ fs and (c)-(d) $t_p = 2.7$ fs. The panel (a) is identical to Fig. 2(c) and the panel (c) is identical to Fig. 2(d) in the main text.

energy $\omega_{\rm in}$. As long as these factors are considerable for all involved states, the signal has time-dependent interference terms related to the dynamics of the system being in the nonstationary state.

For the pulse duration of $\tau_p = 300$ as, this function is much broader than energy differences between eigenenergies ε_1, ε_2 and ε_3 of the involved states. Therefore, the contributions from all involved states are equally considerable and the signal shows a clear time dependence due to the dynamics. For longer pulse durations, starting from about $\tau_p = 700$ as in our case, the function becomes spectrally narrow. It is then impossible to have considerable contributions simultaneously from several states. For this reason, all interference terms would become negligible and the signal would become time-independent.

We calculated the signal for various pulse durations τ_p at fixed incident and scattered photon energies $\omega_{\rm in} = 2490$ eV and $\omega_{\rm s} = 2498.4$ eV, which corresponds to the final state being the ground state. With increasing τ_p, the difference

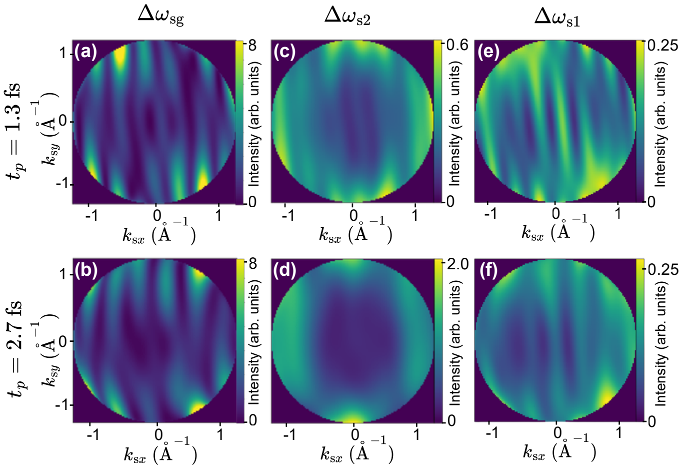

FIG. S4. The detector maps $P(\mathbf{k}_{\rm s}, t_p)$ without the application of the reflective polarizer and for $\mathbf{k}_{\rm in}||z$ with the final state being the ground state ($\omega_{\rm s} - \omega_{\rm in} = \Delta\omega_{\rm sg}$) at (a) $t_p = 1.3$ fs and (b) $t_p = 2.7$ fs. (c)–(f) The same as (a)–(b), but with the final state being a valence-excited state at (c) $\omega_{\rm s} - \omega_{\rm in} = \Delta\omega_{\rm s2}$ and $t_p = 1.3$ fs; (d) $\omega_{\rm s} - \omega_{\rm in} = \Delta\omega_{\rm s2}$ and $t_p = 2.7$ fs; (e) $\omega_{\rm s} - \omega_{\rm in} = \Delta\omega_{\rm s1}$ and $t_p = 1.3$ fs and (f) $\omega_{\rm s} - \omega_{\rm in} = \Delta\omega_{\rm s1}$ and $t_p = 2.7$ fs. The time delays and scattering energies are chosen to be the same as in the main text. Therefore, panels (a) and (b) can be compared with Figs. 2(c) and 2(d); (c) and (d) – with Figs. 2(f) and 2(g); (e) and (f) – with Figs. 2(h) and 2(i).

between the symmetric components of the signal at pump-probe delays $t_p = 1.3$ fs and $t_p = 2.7$ fs gradually diminishes (see Fig. S5(b–i)). For $\tau_p = 700$ as, this difference becomes nearly undetectable (see Fig. S5(h–i)). The signal remains time-dependent up to the pulse duration of 600 as.

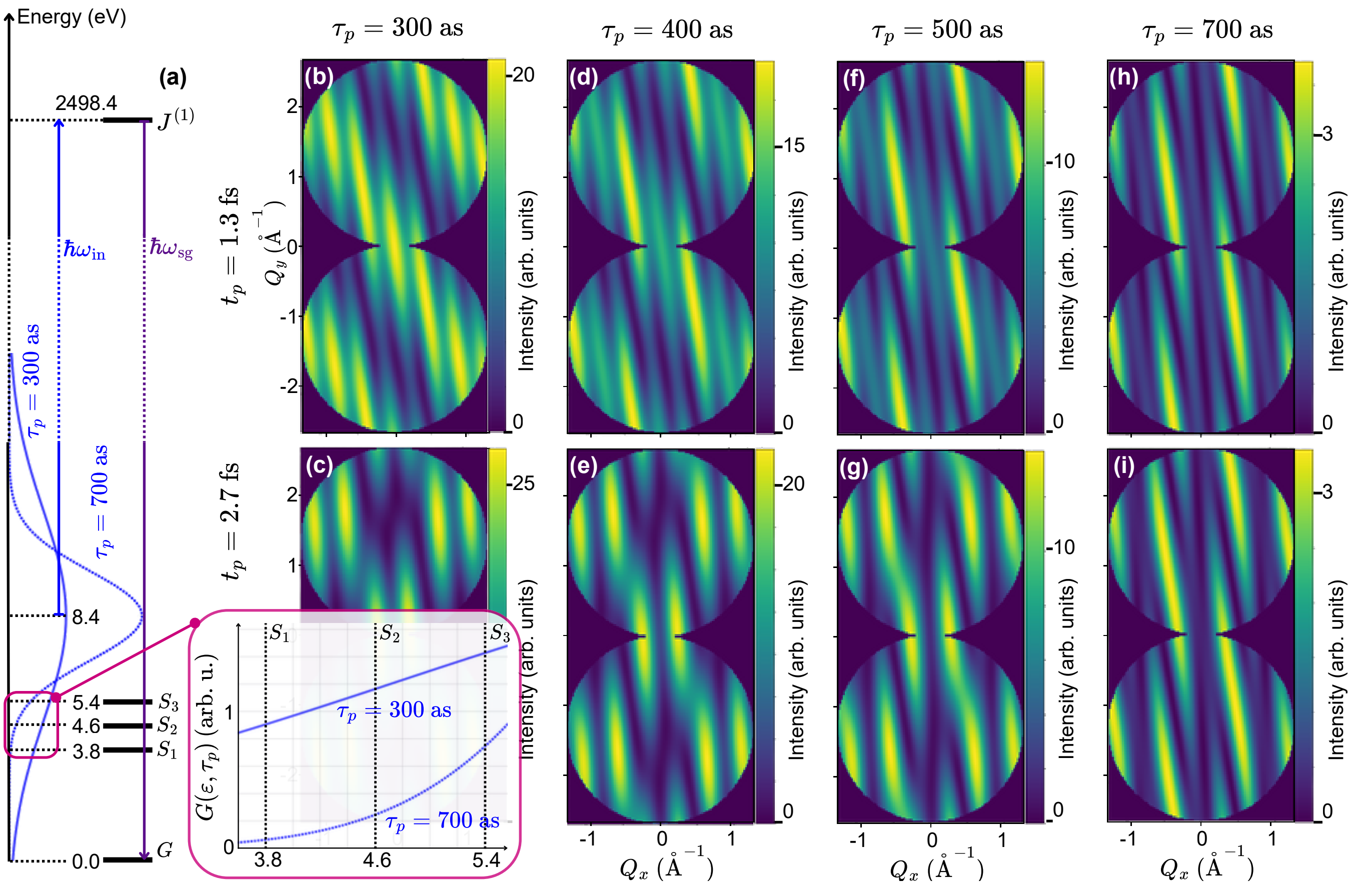

FIG. S5. The signal dependance on the x-ray pulse duration with the final state being the ground state ($\Delta\omega_{\rm sg}$). The scheme of scattering process with pulse-duration-dependent multiplier $G(\varepsilon, \tau_p)$ (a). The symmetric part of the signal with $\tau_p = 300$ as at (a) $t_p = 1.3$ fs and (b) $t_p = 2.7$ fs. (c)–(h) The same as (a)–(b), but with different probe pulse duration (c) $\tau_p = 400$ as and $t_p = 1.3$ fs; (d) $\tau_p = 400$ as and $t_p = 2.7$ fs; (e) $\tau_p = 500$ as and $t_p = 1.3$ fs and (f) $\tau_p = 500$ as and $t_p = 2.7$ fs; (g) $\tau_p = 700$ as and $t_p = 1.3$ fs and (h) $\tau_p = 700$ as and $t_p = 2.7$ fs.

[1] F. Aquilante, J. Autschbach, R. K. Carlson, L. F. Chibotaru, M. G. Delcey, L. De Vico, I. Fdez. Galván, N. Ferré, L. M. Frutos, L. Gagliardi, M. Garavelli, A. Giussani, C. E. Hoyer, G. Li Manni, H. Lischka, D. Ma, P. a. Malmqvist, T. Müller, A. Nenov, M. Olivucci, T. B. Pedersen, D. Peng, F. Plasser, B. Pritchard, M. Reiher, I. Rivalta, I. Schapiro, J. Segarra-Mart ́in, M. Stenrup, D. G. Truhlar, L. Ungur, A. Valentini, S. Vancoillie, V. Veryazov, V. P. Vysotskiy, O. Weingart, F. Zapata, and R. Lindh, Molcas 8: New capabilities for multiconfigurational quantum chemical calculations across the periodic table, Journal of Computational Chemistry **37**, 506 (2016), https://onlinelibrary.wiley.com/doi/pdf/10.1002/jcc.24221.

[2] E. E. Bolton, Y. Wang, P. A. Thiessen, S. H. Bryant, R. A. Wheeler, and D. C. Spellmeyer, Chapter 12 - pubchem: Integrated platform of small molecules and biological activities, in *Annual Reports in Computational Chemistry*, Vol. 4 (Elsevier, 2008) pp. 217–241.

[3] J. Thijssen, The hartree–fock method, in *Computational Physics* (Cambridge University Press, Cambridge, 2007) 2nd ed., pp. 43–88.

[4] K. Pierloot, B. Dumez, P.-O. Widmark, and B. O. Roos, Density matrix averaged atomic natural orbital (ano) basis sets for correlated molecular wave functions, Theoretica chimica acta **90**, 87 (1995).

[5] P. A. Malmqvist, A. Rendell, and B. O. Roos, The restricted active space self-consistent-field method, implemented with a split graph unitary group approach, The Journal of Physical Chemistry **94**, 5477 (1990).

[6] B. S. Fales and T. J. Mart ́innez, Fast transformations between configuration state function and slater determinant bases for direct configuration interaction, The Journal of Chemical Physics **152**, 164111 (2020), https://pubs.aip.org/aip/jcp/article-pdf/doi/10.1063/5.0005155/13418863/164111_1_online.pdf.

[7] V. Sauri, L. Serrano-Andrés, A. R. M. Shahi, L. Gagliardi, S. Vancoillie, and K. Pierloot, Multiconfigurational second-order perturbation theory restricted active space (raspt2) method for electronic excited states: A benchmark study, Journal of Chemical Theory and Computation **7**, 153 (2011).

[8] V. Despré, A. Marciniak, V. Loriot, M. C. E. Galbraith, A. Rouzée, M. J. J. Vrakking, F. Lépine, and A. I. Kuleff, Attosecond hole migration in benzene molecules surviving nuclear motion, The Journal of Physical Chemistry Letters **6**, 426 (2015).

[9] V. Despré, N. V. Golubev, and A. I. Kuleff, Charge migration in propiolic acid: A full quantum dynamical study, Phys. Rev. Lett. **121**, 203002 (2018).

[10] D. T. Matselyukh, V. Despré, N. V. Golubev, A. I. Kuleff, and H. J. Wörner, Decoherence and revival in attosecond charge migration driven by non-adiabatic dynamics, Nature Physics **18**, 1206 (2022).

[11] G. Grell, Z. Guo, T. Driver, P. Decleva, E. Plésiat, A. Picón, J. González-Vázquez, P. Walter, J. P. Marangos, J. P. Cryan, A. Marinelli, A. Palacios, and F. Mart´inn, Effect of the shot-to-shot variation on charge migration induced by sub-fs x-ray free-electron laser pulses, Phys. Rev. Res. **5**, 023092 (2023).

[12] A. Yassar, G. Horowitz, P. Valat, V. Wintgens, M. Hmyène, F. Deloffre, P. Srivastava, P. Lang, and F. Garnier, Exciton coupling effects in the absorption and photoluminescence of sexithiophene derivatives, J. Phys. Chem. **99**, 9155 (1995).

[13] G. Horowitz, S. Romdhane, H. Bouchriha, P. Delannoy, J.-L. Monge, F. Kouki, and P. Valat, Optoelectronic properties of sexithiophene single crystals, Synthetic Metals **90**, 187 (1997).

[14] M. Muccini, Low energy electronic and optical properties of α-sexithiophene single crystals, Mater. Sci. Eng. C **5**, 173 (1998).

[15] S. Möller, G. Weiser, and F. Garnier, Absorption and electroabsorption spectra of an α-sexithiophene single crystal at 20 k, Synthetic Metals **116**, 305 (2001).

[16] B. G. Zhai and Y. M. Huang, Optical properties and electronic structures of sexithiophene, Key Engineering Materials **428–429**, 475 (2010).

[17] T. Zhao, C. Kloc, W. Ni, L. Sun, and G. G. Gurzadyan, Revealing ultrafast relaxation dynamics in six-thiophene thin film and single crystal, J. Photochem. Photobiol. A: Chem. **404**, 112920 (2021).

[18] A. Nenov, F. Segatta, A. Bruner, S. Mukamel, and M. Garavelli, X-ray linear and non-linear spectroscopy of the esca molecule, The Journal of Chemical Physics **151**, 114110 (2019).

[19] D. Popova-Gorelova and R. Santra, Imaging instantaneous electron flow with ultrafast resonant x-ray scattering, Phys. Rev. B **91**, 184303 (2015).
